# Perinatal Posttraumatic Stress Disorder Diagnoses Among Commercially Insured People Increased, 2008–20

**DOI:** 10.1377/hlthaff.2023.01447

**Published:** 2024-04

**Authors:** Stephanie V. Hall, Sarah Bell, Anna Courant, Lindsay K. Admon, Kara Zivin

**Affiliations:** University of Michigan, Ann Arbor, Michigan.; University of Michigan.; University of Michigan.; University of Michigan.; University of Michigan, Veterans Affairs Ann Arbor Healthcare System, and Mathematica, Ann Arbor, Michigan.

## Abstract

Posttraumatic stress disorder (PTSD) is a burdensome disorder, affecting 3–4 percent of delivering people in the US, with higher rates seen among Black and Hispanic people. The extent of clinical diagnosis remains unknown. We describe the temporal and racial and ethnic trends in perinatal PTSD diagnoses among commercially insured people with live-birth deliveries during the period 2008–20, using administrative claims from Optum’s Clinformatics Data Mart Database. Predicted probabilities from our logistic regression analysis showed a 394 percent increase in perinatal PTSD diagnoses, from 37.7 per 10,000 deliveries in 2008 to 186.3 per 10,000 deliveries in 2020. White people had the highest diagnosis rate at all time points (208.0 per 10,000 deliveries in 2020), followed by Black people, people with unknown race, Hispanic people, and Asian people (188.7, 171.9, 146.9, and 79.8 per 10,000 deliveries in 2020, respectively). The significant growth in perinatal PTSD diagnosis rates may reflect increased awareness, diagnosis, or prevalence of the disorder. However, these rates fall well below the estimated prevalence of PTSD in the perinatal population.

Posttraumatic stress disorder (PTSD) is a burdensome mental health disorder affecting roughly 3–4 percent of delivering people,^1^ with higher rates seen among Black and Hispanic people.^2^ PTSD is an anxiety disorder^3^ that occurs in response to trauma, characterized by symptoms of intrusive memories of the traumatic incident, avoidance, negative mood, and increased arousal.^4^ PTSD symptoms typically develop within three months of the precipitating trauma, and symptoms range from mild to severe.^5^

Perinatal PTSD includes PTSD episodes occurring during pregnancy through one year postpartum. Among delivering people, perinatal PTSD negatively affects physical and mental health,^6,7^ interpersonal relationships,^8^ and parenting capacity.^9^ PTSD and traumatic stress responses have intergenerational consequences for the mother, the infant, and their family members.^10^

Risk factors for perinatal PTSD include obstetric factors such as severe maternal morbidity^11^ and preeclampsia,^12^ comorbid psychiatric diagnoses,^13^ social factors,^14^ and distressing or traumatic delivery.^15^ In a study published in 2014, about half of delivering people described their births as traumatic;^16^ in another study published in 2004, up to 10 percent reported experiencing traumatic stress response (a response that includes intrusion and avoidance symptoms but does not meet full diagnostic criteria for PTSD).^17^

Although traumatic pregnancy and delivery are well-studied sources of perinatal PTSD, the precipitating trauma of perinatal PTSD may be unrelated to pregnancy or birth^18^ and may occur at any time before or after pregnancy. Pregnancy and delivery might or might not retrigger PTSD symptoms.^18^ Research indicates that people with prepregnancy PTSD whose symptoms increase during the perinatal period may be especially vulnerable to other maternal mental health conditions.^19^

High comorbidity between PTSD and other mental health disorders is particularly relevant to the delivering population because perinatal mood and anxiety disorders (PMAD) represent the most common pregnancy-related complication; a study published in 2005 found that it affected one in five delivering women.^20^ Prior literature indicates that PMAD rates increased dramatically between 2006 and 2015,^21^ likely the result of increased awareness; screening recommendations; and policy shifts that increased access to mental health care, such as the Mental Health Parity and Addiction Equity Act of 2008 and the Affordable Care Act (ACA) of 2010.^22^ These same factors may have also influenced PTSD diagnosis rates.

Racial and ethnic disparities remain a pressing concern in maternal mental health care.^23^ Several studies demonstrate that Black and Hispanic pregnant women screened positive for PTSD symptoms at higher rates than White pregnant women,^2,24^ in part as a result of higher rates of stressful and traumatic life events among women who are members of racial and ethnic minority groups.^25^ However, such women also face additional barriers to adequate mental health care,^26^ which may delay or prevent the diagnosis of PTSD.

The estimated prevalence of perinatal PTSD of 3–4 percent was based on community samples and cohort studies that actively screened perinatal people for PTSD, using validated assessment tools.^1,18^ Neither prenatal nor postpartum care includes routine screening for PTSD or specifically assesses PTSD risk. As a consequence, perinatal PTSD may be underdiagnosed in real-world settings. The real-world perinatal PTSD diagnosis rate represents the percentage of patients who have been diagnosed with PTSD by a clinician, and this rate remains unknown. The diagnosis rate represents clinically identified PTSD cases, as opposed to prevalence, which represents all PTSD cases. Perinatal PTSD diagnosis rates that are significantly lower than perinatal PTSD prevalence may indicate underdiagnosis and warrant additional research to better identify, diagnose, and treat perinatal PTSD.

This study aims to fill a critical knowledge gap by describing trends in annual perinatal PTSD diagnosis rates among commercially insured people who delivered between 2008 and 2020, both overall and by race and ethnicity. We hypothesized that perinatal PTSD diagnosis rates would increase over the course of the study period as a result of clinical, cultural, and policy shifts related to maternal mental health. We hypothesized that perinatal PTSD diagnosis rates would be lower among delivering people who were members of racial and ethnic minority groups because of historic and structural disparities in mental health care. We hypothesized that perinatal PTSD diagnoses would be concentrated among those with PMAD diagnoses as a result of high levels of psychiatric comorbidity.

## Study Data And Methods

### DATA

In this serial, cross-sectional study, we used deidentified administrative claims data from Optum’s Clinformatics Data Mart Database to determine the annual rate of perinatal PTSD diagnoses among commercially insured people who had a documented live-birth delivery during the period 2008–20. These administrative claims contain comprehensive diagnosis and utilization records for a national, commercially insured population, including people from all fifty US states and Washington, D.C. Optum provides additional information regarding the Clinformatics Data Mart Database data online.^27^

We included people coded as female, ages 15–44, and with a diagnosis or procedural code indicating a live birth between 2008 and 2020. In 2020, for example, we identified 10,175,967 enrollees coded as female in the Optum Database. Once sex, age, and delivery criteria were met, we found that 58 percent of deliveries were among people who were continuously enrolled for one year before and after delivery.

We identified delivery hospitalizations with live births using International Classification of Diseases, Clinical Modification (ICD-9/10-CM), diagnosis and procedure codes. We included only people with continuous enrollment in a single health plan during the calendar year before and after delivery to ensure equal observation of all enrollees. The codes used to identify delivery hospitalizations with live birth appear in supplemental exhibit 1 in the online appendix.^28^

The dependent variable of interest was perinatal PTSD diagnosis, which we defined as any PTSD diagnosis on claims up to one calendar year before and after delivery, similar to the accepted definition of the broader category of perinatal mental illness.^29^ We required the PTSD diagnosis to appear in either two outpatient claims or one inpatient claim, a common approach used to avoid including spurious misdiagnoses. We included PTSD diagnoses appearing in any diagnosis field (not just the primary reason or visit diagnosis field). The ICD-9/10-CM diagnosis codes used to identify PTSD diagnoses appear in supplemental exhibit 2.^28^

The key independent variables of interest were race and ethnicity and delivery year. The race and ethnicity variable included categories of Asian, Black, Hispanic, White, and unknown or missing. We used delivery year to assess temporal changes and defined it as a discrete numeric variable indexed to the year of the delivery hospitalization claim. The unit of analysis was the delivery.

Covariates included age, comorbidities, and income as a percentage of the federal poverty level. We categorized age as 15–26, 27–34, 35–39, and 40 or older. We used the Bateman Obstetric Comorbidity Index to measure comorbidities.^30^ The index is a composite score of perinatal risk factors such as age, body mass index, and comorbid diagnoses. The Bateman Obstetric Comorbidity Index score range is 0–45. Higher scores reflect more medically complex patients at greater risk for complications or poor outcomes. Bateman Obstetric Comorbidity Index scores skew right, and scores rarely exceed 15; the median score is 2.^31^ We dichotomized Bateman Obstetric Comorbidity Index scores at the median observed in our data as 0–1 or 2 or higher.

### ANALYSIS

We derived the federal poverty level using household income and the number of children and adults in the household from the Clinformatics Data Mart Database. We used the federal poverty level guidelines from the Department of Health and Human Services Office of the Assistant Secretary for Planning and Evaluation and the *Federal Register* for each household size to calculate and categorize delivering people as those with incomes of less than 250 percent of poverty, 250–400 percent of poverty, more than 400 percent of poverty, or unknown. These cut-offs reflect common thresholds for additional assistance with health care tax credits, subsidies, and cost sharing.

We used PMAD diagnosis as a stratifying variable.We defined PMAD as any diagnosis of mood or anxiety disorder in two outpatient claims or one inpatient claim in the calendar year before or after delivery. The ICD-9/10-CM codes we used to identify PMAD appear in supplemental exhibit 3.^28^

We applied logistic regression analysis to assess the odds of any PTSD diagnosis during the perinatal period among the cohort of delivering people who met our inclusion criteria. In unadjusted analyses, we included only race and ethnicity and delivery year. In adjusted analyses, we controlled for age, comorbidities (Bateman Obstetric Comorbidity Index score), and income level. We used the predicted probabilities from the adjusted model to examine the annual perinatal PTSD diagnosis rates overall and by race and ethnicity (presented as rates per 10,000 deliveries). In addition, we examined stratified predicted probabilities to determine annual PTSD diagnosis rates among those with and without a comorbid PMAD diagnosis. We coded missing values as “unknown” and included them as separate categories in our analysis for the race and ethnicity and federal poverty level variables, so that we could include as many people as possible to reduce bias. No other variables had missing values. We conducted data management and analysis in SAS, version 9.4. Our use of deidentified secondary data was exempt from Institutional Review Board review.

### LIMITATIONS

Our study had several limitations. Our analytic cohort included only continuously enrolled, privately insured people and might not generalize to the larger delivering population. The study period included the years before and after major infrastructural changes, including the transition from ICD-9-CM to ICD-10-CM, and broader societal changes that this study could not quantitatively measure. We do not know the true prevalence of perinatal PTSD among the study cohort, nor do we have data regarding PTSD screening. The race and ethnicity variable was derived from a proprietary Optum algorithm, which assigns race based on factors such as name and geographic location.^32^ This algorithm has not been validated and might not reflect patient-reported race and ethnicity^32^ or the composition of the general population. The level of unknown race and ethnicity was similar to that found in other observational health research,^33^ but these unknown values limited our ability to comprehensively describe perinatal PTSD across racial and ethnic groups. This analysis only included pregnancies that resulted in live births because of inadequate data regarding other pregnancy outcomes.

## Study Results

We identified 621,148 continuously insured people with a live-birth delivery during the period 2008–20 (*N* = 736,325 deliveries) (data not shown). Sociodemographic characteristics of delivering people changed over the course of the study period. People who delivered in 2020 were slightly older, had more comorbidities including PMAD diagnoses, and had lower incomes compared with people who delivered in 2008; however, the racial and ethnic composition of delivering people remained relatively stable during the period.

During the period 2008–20, a total of 6,075 (0.8 percent) deliveries had a diagnosis of PTSD during the perinatal period. The prevalence of perinatal PTSD diagnoses increased dramatically over the course of the study period. The cohort included 240 (0.4 percent) perinatal PTSD diagnoses among 63,710 deliveries in 2008 and 954 (1.9 percent) perinatal PTSD diagnoses among 51,220 deliveries in 2020 (data not shown).

The sociodemographic characteristics of people with perinatal PTSD also changed over the course of the study period. By 2020, delivering people with perinatal PTSD diagnoses had more comorbidities, including PMAD diagnoses; had lower incomes; and were more likely to be members of racial and ethnic minority groups compared with delivering people with perinatal PTSD diagnoses in 2008. The age of delivering people with perinatal PTSD diagnoses fluctuated over the course of the study period, without a clear trend. Sociodemographic characteristics of people with and without perinatal PTSD diagnoses who delivered in 2008 and 2020 appear in exhibit 1, and all delivery years appear in supplemental exhibits 4 and 5.^28^

In unadjusted logistic regression, accounting only for temporal and racial and ethnic trends, the odds of perinatal PTSD diagnosis increased over the course of the study period. The odds of perinatal PTSD diagnosis remained relatively stable from 2008 to 2010, before progressively increasing from 2011 to 2020. By 2020, the odds of diagnosis were greater than in 2008 (odds ratio: 5.02). Delivering people who were members of racial and ethnic minority groups experienced lower odds of perinatal PTSD diagnosis compared with White delivering people. Black people, Hispanic people, and people of unknown race and ethnicity had lower odds of PTSD diagnosis than White people (OR: 0.90, 0.70, and 0.81, respectively). Asian people had the lowest odds of perinatal PTSD diagnosis compared with White people (OR: 0.37). The unadjusted logistic regression model of perinatal PTSD diagnosis among delivering people appears in exhibit 2.

These trends remained similar after we adjusted for age, comorbidities, and income level. As with unadjusted analyses, the adjusted odds of diagnosis remained relatively stable from 2008 to 2010 but grew consistently from 2011 to 2020. By 2020, the adjusted odds of perinatal PTSD diagnosis were greater than in 2008 (adjusted OR: 4.81). Compared with White people, the adjusted odds of perinatal PTSD diagnosis were lower for Asian people, Black people, Hispanic people, and people of unknown race and ethnicity (aOR: 0.39, 0.77, 0.63, and 0.72, respectively).

Several sociodemographic characteristics were significantly associated with perinatal PTSD diagnosis. People ages 15–26 had greater odds of perinatal PTSD diagnosis compared with people age forty or older (aOR: 2.16). The adjusted odds of perinatal PTSD diagnosis were not significantly different for people ages 27–34 or 35–39 compared with those age forty or older. People with more comorbidities, as measured by a Bateman Obstetric Comorbidity Index score of 2 or higher, had greater odds of perinatal PTSD diagnosis than people with scores of 0–1 (aOR: 1.85). Finally, compared to people with incomes higher than 400 percent of poverty, people with incomes lower than 250 percent of poverty, people with incomes of 250–400 percent of poverty, and people with unknown poverty levels had greater adjusted odds of perinatal PTSD diagnosis (aOR: 1.40, 1.20, and 1.34, respectively). The adjusted logistic regression model of perinatal PTSD diagnoses among delivering people appears in exhibit 2, and a forest plot visually displays this model in supplemental exhibit 6.^28^

We used the adjusted logistic regression model to produce the annual perinatal PTSD diagnosis rate per 10,000 deliveries overall and by race and ethnicity. The predicted probability of perinatal PTSD diagnosis increased dramatically from 2008 to 2020 both overall and among all races and ethnicities. The overall predicted probability of perinatal PTSD diagnosis increased 394 percent, from 37.7 perinatal PTSD diagnoses per 10,000 deliveries in 2008 to 186.3 perinatal PTSD diagnoses per 10,000 deliveries in 2020 (data not shown).

Although the predicted probability of perinatal PTSD diagnosis increased for all races and ethnicities, disparities persisted throughout the study period. From 2008 to 2020, the predicted probability of perinatal PTSD diagnosis rose from 42.2 to 208.0 per 10,000 deliveries among White people, from 37.6 to 188.7 per 10,000 deliveries among Black people, from 36.1 to 171.9 per 10,000 deliveries among people of unknown race, from 29.2 to 146.9 per 10,000 deliveries among Hispanic people, and from 15.0 to 79.8 per 10,000 deliveries among Asian people. Visual inspection of these rates reveals a steady but gradual increase in PTSD diagnosis rates from 2008 to 2013, followed by a more dramatic annual increase from 2014 to 2020 for all races and ethnicities. The annual predicted probability of perinatal PTSD diagnosis by race and ethnicity appears in exhibit 3.

We used the same adjusted logistic regression model to produce annual perinatal PTSD diagnosis rates stratified by PMAD diagnosis, which indicate that the increase in PTSD diagnosis rates was concentrated among people with PMAD diagnoses. This regression model is shown in supplemental exhibit 7 and visually displayed in supplemental exhibit 8.^28^ From 2008 to 2020, the predicted probability of PTSD diagnosis among people without PMAD diagnoses rose from 13.4 per 10,000 deliveries to 35.3 per 10,000 deliveries. During the same period, the predicted probability of perinatal PTSD diagnosis among people with PMAD diagnoses rose from 180.5 per 10,000 deliveries to 571.4 per 10,000 deliveries. The annual adjusted predicted probability of perinatal PTSD diagnosis by PMAD diagnosis appears in exhibit 4.

## Discussion

Perinatal PTSD diagnoses among commercially insured delivering people increased dramatically over the course of a twelve-year period, 2008–20. Rates of diagnosis varied by race and ethnicity, with White people having the highest diagnosis rate across all time points, followed by Black people, people with unknown race and ethnicity, Hispanic people, and Asian people, even after other factors were adjusted for.

The observed perinatal PTSD diagnosis rates represent the prevalence of diagnosis, distinct from the prevalence of disease. Most existing literature assesses the prevalence of disease, but one study assessed self-report of PTSD diagnosis among a convenience sample of obstetric patients in the midwestern US and found a self-reported diagnosis rate of 7.9 percent during the perinatal period.^25^ The highest diagnosis rate observed in our study was 2.1 percent, seen among White people in 2020. The comparatively low rate in our study may be a result of methodologic differences related to convenience sampling and self-report, or it may indicate that commercially insured delivering people have lower rates of perinatal PTSD compared with the general US population.

Comparing diagnosis rates with population estimates of prevalence offers evidence regarding whether routine care adequately diagnoses cases at the population level, which is an important measure, as diagnosis typically indicates the first step toward treatment and remission. One meta-analysis estimated that 3.3 percent of pregnant people and 4.0 percent of postpartum people experienced PTSD in global settings,^1^ and another meta-analysis estimated that 3.1 percent of postpartum people experienced PTSD in global settings.^18^ The observed diagnosis rates in our study fall below these estimates of prevalence. This may be because of methodologic differences, as most existing literature specifically screened for perinatal PTSD to assess prevalence, whereas our work aimed to measure real-world diagnosis rates established through routine clinical care. Underdiagnosis of maternal mental health disorders, including PMAD and postpartum depression, is a well-studied and pervasive problem,^34^ attributed to clinical, financial, and social barriers to care.^22,34,35^ These same barriers may result in underdiagnosis of perinatal PTSD. In addition, commercially insured delivering people in the US may have lower rates of perinatal PTSD than global populations.

The increase in perinatal PTSD diagnoses over the course of the study period may reflect increased awareness, diagnosis, or prevalence of the disorder or a combination of all three. Multiple major cultural and policy changes occurred during this period. The Mental Health Parity and Addiction Equity Act required insurance plans to provide mental health benefits at a level equal to medical and surgical benefits, and the Affordable Care Act required insurance plans to cover ten essential health benefits, including preventive and maternal care.^22^ In 2015, the American College of Obstetricians and Gynecologists recommended universal screening of pregnant and postpartum people for perinatal depression,^36^ and in 2016, the U.S. Preventive Services Task Force issued a similar recommendation.^37^ Together, these changes aimed to improve maternal mental health care^22,36^ and may have played a role in increasing rates of perinatal PTSD diagnosis.

Our results indicate that delivering people who are members of racial and ethnic minority groups were less likely than White delivering people to be diagnosed with perinatal PTSD. In the aforementioned study of self-reported diagnosis among midwestern US obstetric patients,^25^ African American people self-reported higher diagnosis rates than non–African American people (13.4 percent versus 3.5 percent). Our study’s observed racial and ethnic diagnosis rates cannot be directly compared with the results from that study because of the use of different racial and ethnic categories. Most literature indicates that delivering people who are members of racial and ethnic minority groups experience perinatal PTSD symptoms at higher rates than White delivering people,^2,19,25^ although one study of postpartum White, Asian, and Pacific Islander people found no significant racial and ethnic disparities in PTSD symptoms.^38^

Our results may indicate racial and ethnic disparities in diagnosis. This interpretation would be consistent with literature on such disparities in the diagnosis of other maternal mental health disorders. For example, Black Medicaid enrollees with PMAD symptoms are less likely to be diagnosed with PMAD than White Medicaid enrollees with PMAD symptoms.^39^ Black people are more likely to face individual and systemic barriers to mental health care than White people, such as culturally insensitive care, fear of disclosing symptoms, stigma, and financial barriers.^40^ In addition, evidence suggests that PTSD symptoms manifest differentially among different races and ethnicities,^2^ which may delay or prevent diagnosis among some subgroups.

The high rate of perinatal PTSD diagnoses among people with PMAD diagnoses is consistent with our hypothesis and the literature regarding the high rate of co-occurrence of PTSD and depression among other populations. A meta-analysis estimated that 44.1 percent (95% confidence interval: 34.4, 54.2) of pregnant people with PTSD also experienced depression, and 17.7 percent (95% CI: 1.9, 70.3) of postpartum people with PTSD also experienced depression.^1^ The large confidence intervals reflect a dearth of literature on mental health comorbidities among delivering people with PTSD.

Policies that promote appropriate PTSD diagnosis may facilitate treatment and improve outcomes.We recommend that providers and policy makers develop evidence-based PTSD screening guidelines, provide perinatal PTSD training and resources for clinicians, ensure that payment incentives align with PTSD screening guidelines, and invest in culturally sensitive practices to mitigate disparities.

Evidence-based PTSD screening guidelines would provide a standardized approach to PTSD management. The American College of Obstetricians and Gynecologists^36^ and the U.S. Preventive Services Task Force^37^ endorsed universal screening to improve detection of perinatal depression.^41^ PTSD screening guidelines could similarly improve the detection of perinatal PTSD.^24^ Organizations such as these should study, develop, and issue evidence-based PTSD screening guidelines.

Obstetricians represent the primary point of care for perinatal people. However, only 11 percent of obstetrics-gynecology residency programs cover PTSD.^42^ Increased funding of continuing education and provider-facing resources such as Psychiatry Access Programs, a government-funded psychiatric consultation service for maternal health care providers,^43^ would fill gaps in providers’ knowledge.

Payment incentives must align with appropriate PTSD screening guidelines. Obstetric care is typically “bundled” and might not incentivize mental health screening.^44^ Payers should explore alternative models to incentivize appropriate PTSD care through screening reimbursement or value-based care. This may also encourage documentation of screening, which would create more robust data regarding screening patterns.

Investing in culturally sensitive, trauma-informed care is critical to reducing disparities and tailoring PTSD management to racial and ethnic minority populations. Because disparities in PTSD result from disparities in exposure to trauma,^2,19,25^ funding antiracist initiatives to reduce poverty, violence, and structural racism in racial and ethnic minority communities may mitigate disparities through prevention.

In addition to policy changes, additional research is needed. Our work has evaluated the prevalence of perinatal PTSD diagnoses. However, the onset of PTSD may differ markedly from the time of PTSD diagnosis. Future research should assess PTSD diagnosis at different time points during the perinatal period, the time between symptom onset and diagnosis, and the heterogeneity of timely perinatal PTSD diagnosis among racial and ethnic groups.

## Conclusion

Perinatal PTSD diagnoses increased significantly over the course of the study period, and substantial disparities in detection exist. Diagnosis rates might not reflect the true prevalence of PTSD in the population of delivering people. Providers and policy makers should work to ensure appropriate screening, diagnosis, and treatment for all delivering people suffering from PTSD to mitigate the personal, family, and generational impacts of the condition.

## Supplementary Material

Appendix

Video Abstract

## Figures and Tables

**EXHIBIT 3:**
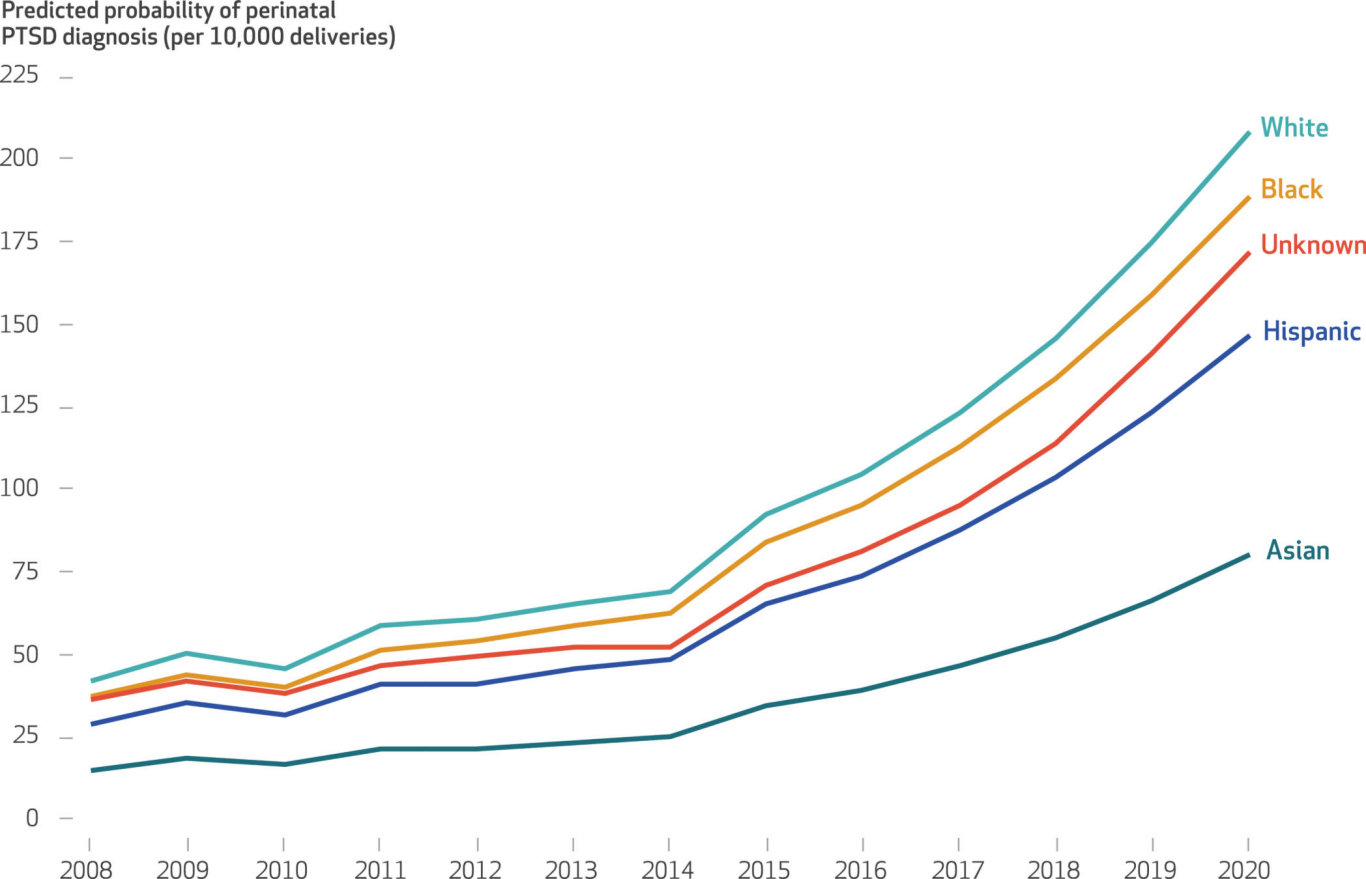
Annual adjusted predicted probability of perinatal posttraumatic stress disorder (PTSD) diagnosis per 10,000 deliveries among US commercially insured delivering people, by race and ethnicity, 2008–20 **SOURCE** Authors’ analysis of Optum Clinformatics Data Mart claims data for women ages 15–44 who had a live-birth delivery during 2008–20 and had continuous enrollment in a single health plan for 12 months before and 12 months after delivery. **NOTE** This figure presents the results of an adjusted logistic regression model as described in the text and exhibit 2.

**EXHIBIT 4:**
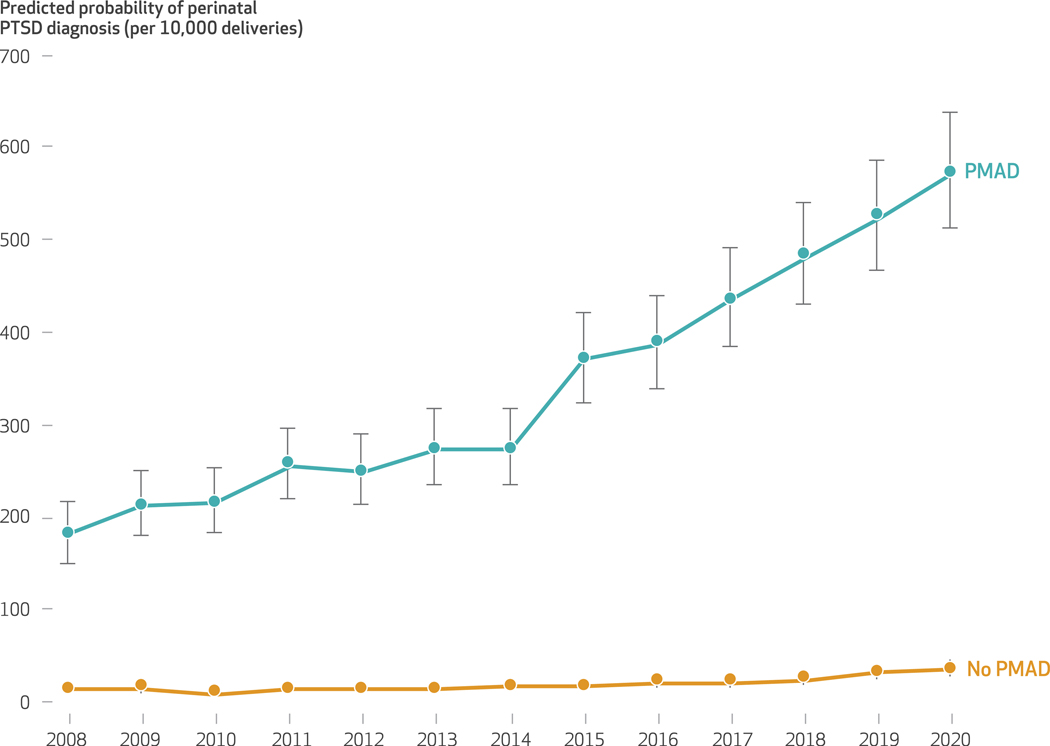
Annual adjusted predicted probability of perinatal posttraumatic stress disorder (PTSD) diagnosis per 10,000 deliveries among US commercially insured delivering people with and without perinatal mood and anxiety disorder (PMAD) diagnosis, 2008–20 **SOURCE** Authors’ analysis of Optum Clinformatics Data Mart claims data for women ages 15–44 who had a live-birth delivery during 2008–20 and had continuous enrollment in a single health plan for 12 months before and 12 months after delivery. **NOTES** This figure presents the results of an adjusted logistic regression model as described in supplemental exhibits 7 and 8 (see note 28 in text). Whiskers indicate 95% confidence intervals.

**EXHIBIT 1:** Sociodemographic characteristics of US commercially insured delivering people with and without posttraumatic stress disorder (PTSD) diagnoses, 2008 and 2020

	2008				2020			
	PTSD (*n* = 240)		No PTSD (*n* = 63,470)		PTSD (*n* = 954)		No PTSD (*n* = 50,266)	
	Number	Percent	Number	Percent	Number	Percent	Number	Percent
Race and ethnicity Asian	—^a^	—^a^	4,223	6.7	32	3.4	3,364	6.7
Black	15	6.3	5,681	9.0	94	9.9	4,137	8.2
Hispanic	16	6.7	7,941	12.5	103	10.8	6,390	12.7
Unknown	—^a^	—^a^	6,935	10.9	85	8.9	5,419	10.8
White	178	74.2	38,690	61.0	640	67.1	30,956	61.6
Age, years								
15–26	60	25.0	10,965	17.3	263	27.6	7,181	14.3
27–34	102	42.5	34,176	53.8	437	45.8	27,051	53.8
35–39	52	21.7	14,452	22.8	202	21.2	12,948	25.8
40 or older	26	10.8	3,877	6.1	52	5.5	3,086	6.1
OBCMI score								
0–1	173	72.1	49,917	78.6	634	66.5	37,149	73.9
2 or higher	67	27.9	13,553	21.4	320	33.5	13,117	26.1
PMAD diagnosis								
No	73	30.4	54,387	85.7	130	13.6	36,669	72.9
Yes	167	69.6	9,083	14.3	824	86.4	13,597	27.1
Income as percent of federal poverty level								
Less than 250%	22	9.2	7,224	11.4	283	29.7	11,818	23.5
250–400%	43	17.9	9,770	15.4	247	25.9	12,971	25.8
More than 400%	112	46.7	31,977	50.4	292	30.6	17,876	35.6
Unknown	63	26.3	14,499	22.8	132	13.8	7,601	15.1

**SOURCE** Authors’ analysis of Optum Clinformatics Data Mart claims data for women ages 15–44 who had a live-birth delivery during 2008–20 and had continuous enrollment in a single health plan for 12 months before and 12 months after delivery. **NOTES** OBCMI is Bateman Obstetric Comorbidity Index. PMAD is perinatal mood and anxiety disorder.

a Cell sizes lower than 11 are suppressed.

**EXHIBIT 2:** Unadjusted and adjusted odds of posttraumatic stress disorder (PTSD) diagnosis among US commercially insured delivering people, 2008–20

	Unadjusted odds ratio	95% CI	*p* value	Adjusted odds ratio	95% CI	*P* value
Race and ethnicity						
Asian	0.37	0.32, 0.43	<0 0001	0.39	0.34, 0.45	<0 0001
Black	0.90	0.83, 0.99	0.0281	0.77	0.70, 0.84	<0 0001
Hispanic	0.70	0.65, 0.76	<0 0001	0.63	0.58, 0.68	<0 0001
Unknown	0.81	0.72, 0.91	0.0005	0.72	0.63, 0.82	<0 0001
White	Ref	Ref	Ref	Ref	Ref	Ref
Delivery year						
2008	Ref	Ref	Ref	Ref	Ref	Ref
2009	1.19	1.00, 1.41	0.048	1.18	1.00, 1.41	0.0564
2010	1.08	0.91, 1.29	0.3844	1.07	0.90, 1.28	0.4260
2011	1.38	1.17, 1.64	0.0002	1.36	1.15, 1.62	0.0003
2012	1.42	1.20, 1.68	<0 0001	1.38	1.16, 1.63	0.0002
2013	1.54	1.30, 1.82	<0 0001	1.48	1.25, 1.76	<0 0001
2014	1.63	1.38, 1.93	<0 0001	1.58	1.34, 1.87	<0 0001
2015	2.19	1.87, 2.57	<0 0001	2.11	1.80, 2.47	<0 0001
2016	2.48	2.13, 2.90	<0 0001	2.39	2.04, 2.79	<0 0001
2017	2.95	2.53, 3.43	<0 0001	2.83	2.43, 3.29	<0 0001
2018	3.49	3.02, 4.05	<0 0001	3.33	2.87, 3.86	<0 0001
2019	4.19	3.63, 4.84	<0 0001	3.99	3.45, 4.62	<0 0001
2020	5.02	4.35, 5.79	<0.0001	4.81	4.17, 5.54	<0.0001
Age, years						
15–26	—^a^	—^a^	—^a^	2.16	1.92, 2.43	<0 0001
27–34	—^a^	—^a^	—^a^	1.03	0.92, 1.16	0.5720
35–39	—^a^	—^a^	—^a^	1.06	0.95, 1.19	0.2853
40 or older	—^a^	—^a^	—^a^	Ref	Ref	Ref
OBCMI score						
0–1	—^a^	—^a^	—^a^	Ref	Ref	Ref
2 or higher	—^a^	—^a^	—^a^	1.85	1.74, 1.96	<0.0001
Income as percent of federal poverty level						
Less than 250%	—^a^	—^a^	—^a^	1.40	1.30, 1.50	<0 0001
250–400%	—^a^	—^a^	—^a^	1.20	1.12, 1.29	<0 0001
More than 400%	—^a^	—^a^	—^a^	Ref	Ref	Ref
Unknown	—^a^	—^a^	—^a^	1.34	1.23, 1.45	<0.0001

**SOURCE** Authors’ analysis of Optum Clinformatics Data Mart claims data of women ages 15–44 who had a live-birth delivery during 2008–20 and had continuous enrollment in a single health plan for 12 months before and 12 months after delivery. **NOTES** Reference value is 1.00. Additional details are in the technical appendix (see note 28 in text). OBCMI is Bateman Obstetric Comorbidity Index.

a No adjustment was made in the model for the covariate.
