# Supplementary material for: Perinatal Posttraumatic Stress Disorder Diagnoses Among Commercially Insured People Increased, 2008–20
Source: Health Aff (Millwood). Author manuscript; Available in PMC 2024 Jul 5. (PMC11225106; doi:10.1377/hlthaff.2023.01447)
Supplement: Appendix [file NIHMS1996632-supplement-Appendix.pdf]

Hall SV, Bell S, Courant A, Admon LK, Zivin K.
Perinatal posttraumatic stress disorder diagnoses among commercially insured people increased, 2008–20.
Health Aff (Millwood). 2024;43(4).

Appendix

Appendix Exhibits

Supplemental Exhibit 1. Codes used to identify delivery hospitalizations for live birth.

Supplemental Exhibit 2. Codes used to identify PTSD diagnoses.

Supplemental Exhibit 3. Codes used to identify PMAD diagnoses.

Supplemental Exhibit 4. Sociodemographic characteristics of delivering individuals with PTSD 2008-2020.

Supplemental Exhibit 5. Sociodemographic characteristics of delivering individuals without PTSD 2008-2020.

Supplemental Exhibit 6. Forest plot of adjusted odds of PTSD diagnosis.

Supplemental Exhibit 7. Adjusted odds of PTSD diagnosis among those with and without PMAD.

Supplemental Exhibit 8. Forest plot of adjusted odds of PTSD diagnosis among those (a) with and (b) without PMAD.

Technical Appendix

Multivariate Logistic Regression

Supplemental Exhibit 1. Codes used to identify delivery hospitalizations for live birth.

Included: Cesarean and vaginal delivery

	Cesarean delivery	Vaginal delivery
ICD-9-CM procedures	740, 741, 742, 744, 7499	
ICD-10-CM procedures	10D00Z0, 10D00Z1, 10D00Z2	10D07Z3, 10D07Z4, 10D07Z5, 10D07Z6, 10D07Z7, 10D07Z8, 10E0ZZ
DRG	370, 371, 765, 766, 540, 5401, 5402, 5403, 5404	372, 373, 374, 375, 767 768, 774, 775, 541, 542, 560, 5411, 5412, 5413, 5414, 5421, 5422, 5423, 5424, 5601, 5602, 5603
CPT	59510, 59514, 59515, 59618, 59620, 59622	59400, 59409, 59410, 59610, 59612, 59614

Included: Pre-term birth

ICD 10 diagnoses	O601, O6010, O60100, O60101, O60102, O60103, O60104, O60105, O60109, O6012, O60120, O60121, O60122, O60123, O60124, O60125, O60129, O6013, O60130, O60131, O60132, O60133, O60134, O60135, O60139, O6014, O60140, O60141, O60142, O60143, O60144, O60145, O60149
ICD 9 diagnoses	64420, 64421

Excluded: abortion, dilatation and curettage, and stillbirth

ICD procedures and CPT codes	Removal of ectopic pregnancy	59120, 59121, 59130, 59135, 59136, 59140, 59150, 59151, 743
	Abortion (termination of pregnancy)	59840, 59841, 59850, 59851, 59852, 59855, 59856, 59857, 10A00ZZ, 10A03ZZ, 10A04ZZ, 10A07Z, 10A07ZZ, 10A07ZZ, 10A08ZZ, 10A08ZZ, 6901, 6951, 7491, 750
	Dilatation and curettage (D&C); aspiration after delivery or abortion	59812, 59820, 59821, 59830

ICD 9 and 10 diagnoses	Removal of ectopic pregnancy	O0000, O0001, O00101, O00102, O00109, O00111, O00112, O00119, O00201, O00202, O00209, O00211, O00212, O00219, O0080, O0081, O0090, O0091, 63300, 63301, 63310, 63311, 63320, 63321, 63380, 63381, 63390, 63391,

	Abortion (termination of pregnancy)	O030, O031, O032, O0330, O0331, O0332, O0333, O0334, O0337, O0339, O034, O035, O036, O037, O0380, O0381, O0382, O0383, O0384, O0385, O0386, O0387, O0388, O0389, O039, 63400, 63401, 63402, 63410, 63411, 63412, 63420, 63421, 63422, 63430, 63431, 63432, 63440, 63441, 63442, 63450, 63451, 63452, 63460, 63461, 63462, 63470, 63471, 63472, 63480, 63481, 63482, 63490, 63491, 63492, O045, O045, O045, O046, O046, O046, O047, O047, O047, O0480, O0480, O0480, O0481, O0481, O0481, O0482, O0482, O0482, O0483, O0483, O0483, O0484, O0484, O0484, O0485, O0486, O0487, O0488, O0489, O0489, O0489, O070, O071, O072, O0730, O0731, O0732, O0733, O0734, O0735, O0736, O0737, O0738, O0739, O074, Z332, Z332, Z332, 63500, 63501, 63502, 63510, 63511, 63512, 63520, 63521, 63522, 63530, 63531, 63532, 63540, 63541, 63542, 63550, 63551, 63552, 63560, 63561, 63562, 63570, 63571, 63572, 63580, 63581, 63582, 63590, 63591, 63592, 63600, 63601, 63602, 63610, 63611, 63612, 63620, 63621, 63622, 63630, 63631, 63632, 63640, 63641, 63642, 63650, 63651, 63652, 63660, 63661, 63662, 63670, 63671, 63672, 63680, 63681, 63682, 63690, 63691, 63692, 63700, 63701, 63702, 63710, 63711, 63712, 63720, 63721, 63722, 63730, 63731, 63732, 63740, 63741, 63742, 63750, 63751, 63752, 63760, 63761, 63762, 63770, 63771, 63772, 63780, 63781, 63782, 63790, 63791, 63792, 6380, 6381, 6382, 6383, 6384, 6385, 6386, 6387, 6388, 6389, O080, O081, O082, O083, O084, O085, O086, O087, O0881, O0882, O0883, O0889, O089, 6390, 6391, 6392, 6393, 6394, 6395, 6396, 6398, 6399,
	Other complications	O019, O021, O0289, Z371, Z374, Z377, 630, 6310, 6318, 632, V271, V274, V277, A34

Supplemental Exhibit 2. Codes used to identify PTSD diagnoses.

ICD-9-CM	30981
ICD-10-CM	F4310, F4311, F4312

Supplemental Exhibit 3. Codes used to identify PMAD diagnoses.

Group	ICD Diagnosis Code
Anxiety	300, 308, 313, 293, F06, F40, F41, F42, F43, F48, R45
Depression	311, 296, 300, F32, F33

Supplemental Exhibit 4. Sociodemographic characteristics of delivering individuals with PTSD 2008-2020.

	2008	2009	2010	2011	2012	2013	2014	2015	2016	2017	2018	2019	2020
	(n=240)	(n=280)	(n=252)	(n=315)	(n=327)	(n=316)	(n=326)	(n=440)	(n=502)	(n=592)	(n=710)	(n=821)	(n=954)
Age													
15-26	60 (25%)	75 (26.8%)	60 (23.8%)	82 (26%)	98 (30%)	100 (31.6%)	81 (24.8%)	140 (31.8%)	159 (31.7%)	179 (30.2%)	184 (25.9%)	220 (26.8%)	263 (27.6%)
27-34	102 (42.5%)	115 (41.1%)	122 (48.4%)	152 (48.3%)	133 (40.7%)	124 (39.2%)	136 (41.7%)	175 (39.8%)	203 (40.4%)	237 (40%)	304 (42.8%)	364 (44.3%)	437 (45.8%)
35-39	52 (21.7%)	59 (21.1%)	52 (20.6%)	57 (18.1%)	63 (19.3%)	70 (22.2%)	92 (28.2%)	94 (21.4%)	109 (21.7%)	147 (24.8%)	167 (23.5%)	170 (20.7%)	202 (21.2%)
40+	26 (10.8%)	31 (11.1%)	18 (7.1%)	24 (7.6%)	33 (10.1%)	22 (7%)	17 (5.2%)	31 (7%)	31 (6.2%)	29 (4.9%)	55 (7.7%)	67 (8.2%)	52 (5.5%)
OBCMI Score													
0-1	173 (72.1%)	194 (69.3%)	175 (69.4%)	223 (70.8%)	219 (67%)	221 (69.9%)	211 (64.7%)	292 (66.4%)	320 (63.7%)	388 (65.5%)	450 (63.4%)	526 (64.1%)	634 (66.5%)
2+	67 (27.9%)	86 (30.7%)	77 (30.6%)	92 (29.2%)	108 (33%)	95 (30.1%)	115 (35.3%)	148 (33.6%)	182 (36.3%)	204 (34.5%)	260 (36.6%)	295 (35.9%)	320 (33.5%)
PMAD													
No	73 (30.4%)	77 (27.5%)	42 (16.7%)	64 (20.3%)	70 (21.4%)	61 (19.3%)	72 (22.1%)	69 (15.7%)	89 (17.7%)	91 (15.4%)	99 (13.9%)	125 (15.2%)	130 (13.6%)
Yes	167 (69.6%)	203 (72.5%)	210 (83.3%)	251 (79.7%)	257 (78.6%)	255 (80.7%)	254 (77.9%)	371 (84.3%)	413 (82.3%)	501 (84.6%)	611 (86.1%)	696 (84.8%)	824 (86.4%)
Poverty													
<250%	22 (9.2%)	37 (13.2%)	46 (18.3%)	74 (23.5%)	72 (22%)	62 (19.6%)	65 (19.9%)	92 (20.9%)	122 (24.3%)	162 (27.4%)	219 (30.8%)	247 (30.1%)	283 (29.7%)
250-400%	43 (17.9%)	62 (22.1%)	51 (20.2%)	60 (19%)	73 (22.3%)	63 (19.9%)	69 (21.2%)	91 (20.7%)	95 (18.9%)	181 (30.6%)	230 (32.4%)	241 (29.4%)	247 (25.9%)
>400%	112 (46.7%)	110 (39.3%)	103 (40.9%)	119 (37.8%)	124 (37.9%)	112 (35.4%)	121 (37.1%)	165 (37.5%)	164 (32.7%)	178 (30.1%)	204 (28.7%)	235 (28.6%)	292 (30.6%)
Unknown	63 (26.3%)	71 (25.4%)	52 (20.6%)	62 (19.7%)	58 (17.7%)	79 (25%)	71 (21.8%)	92 (20.9%)	121 (24.1%)	71 (12%)	57 (8%)	98 (11.9%)	132 (13.8%)
Race/ethnicity													
Asian	**	**	**	**	**	**	**	**	21 (4.2%)	21 (3.5%)	20 (2.8%)	23 (2.8%)	32 (3.4%)
Black	15 (6.3%)	17 (6.1%)	26 (10.3%)	29 (9.2%)	23 (7%)	23 (7.3%)	33 (10.1%)	30 (6.8%)	48 (9.6%)	60 (10.1%)	66 (9.3%)	75 (9.1%)	94 (9.9%)
Hispanic	16 (6.7%)	26 (9.3%)	32 (12.7%)	41 (13%)	32 (9.8%)	27 (8.5%)	38 (11.7%)	47 (10.7%)	57 (11.4%)	67 (11.3%)	71 (10%)	76 (9.3%)	103 (10.8%)
Unknown	**	**	**	**	**	**	**	**	14 (2.8%)	15 (2.5%)	18 (2.5%)	56 (6.8%)	85 (8.9%)
White	178 (74.2%)	206 (73.6%)	173 (68.7%)	225 (71.4%)	253 (77.4%)	237 (75%)	228 (69.9%)	341 (77.5%)	362 (72.1%)	429 (72.5%)	535 (75.4%)	591 (72%)	640 (67.1%)

Supplemental Exhibit 5. Sociodemographic characteristics of delivering individuals without PTSD 2008-2020.

	2008	2009	2010	2011	2012	2013	2014	2015	2016	2017	2018	2019	2020
	(n=63470)	(n=62165)	(n=61386)	(n=59964)	(n=60531)	(n=54220)	(n=52862)	(n=53051)	(n=53464)	(n=53272)	(n=53820)	(n=51779)	(n=50266)
Age													
15-26	10,965 (17.3%)	10,385 (16.7%)	10,268 (16.7%)	10,083 (16.8%)	11,199 (18.5%)	10,169 (18.8%)	9,451 (17.9%)	9,335 (17.6%)	9,005 (16.8%)	8,547 (16%)	8,263 (15.4%)	7,571 (14.6%)	7,181 (14.3%)
27-34	34,176 (53.8%)	34,221 (55%)	34,241 (55.8%)	33,429 (55.7%)	33,051 (54.6%)	29,423 (54.3%)	28,864 (54.6%)	28,695 (54.1%)	29,129 (54.5%)	28,827 (54.1%)	28,984 (53.9%)	27,909 (53.9%)	27,051 (53.8%)
35-39	14,452 (22.8%)	13,859 (22.3%)	13,012 (21.2%)	12,665 (21.1%)	12,680 (20.9%)	11,633 (21.5%)	11,717 (22.2%)	12,131 (22.9%)	12,449 (23.3%)	12,893 (24.2%)	13,458 (25%)	13,244 (25.6%)	12,948 (25.8%)
40+	3,877 (6.1%)	3,700 (6%)	3,865 (6.3%)	3,787 (6.3%)	3,601 (5.9%)	2,995 (5.5%)	2,830 (5.4%)	2,890 (5.4%)	2,881 (5.4%)	3,005 (5.6%)	3,115 (5.8%)	3,055 (5.9%)	3,086 (6.1%)
OBCMI Score													
0-1	49,917 (78.6%)	48,720 (78.4%)	47,988 (78.2%)	46,910 (78.2%)	47,510 (78.5%)	42,752 (78.8%)	41,763 (79%)	41,166 (77.6%)	40,974 (76.6%)	40,292 (75.6%)	40,283 (74.8%)	38,343 (74.1%)	37,149 (73.9%)
2+	13,553 (21.4%)	13,445 (21.6%)	13,398 (21.8%)	13,054 (21.8%)	13,021 (21.5%)	11,468 (21.2%)	11,099 (21%)	11,885 (22.4%)	12,490 (23.4%)	12,980 (24.4%)	13,537 (25.2%)	13,436 (25.9%)	13,117 (26.1%)
PMAD													
No	54,387 (85.7%)	52,765 (84.9%)	51,816 (84.4%)	50,385 (84%)	50,447 (83.3%)	45,120 (83.2%)	43,818 (82.9%)	43,392 (81.8%)	43,198 (80.8%)	42,242 (79.3%)	41,757 (77.6%)	39,183 (75.7%)	36,669 (72.9%)
Yes	9,083 (14.3%)	9,400 (15.1%)	9,570 (15.6%)	9,579 (16%)	10,084 (16.7%)	9,100 (16.8%)	9,044 (17.1%)	9,659 (18.2%)	10,266 (19.2%)	11,030 (20.7%)	12,063 (22.4%)	12,596 (24.3%)	13,597 (27.1%)
Poverty													
<250%	7,224 (11.4%)	7,547 (12.1%)	7,760 (12.6%)	8,323 (13.9%)	8,895 (14.7%)	8,079 (14.9%)	9,202 (17.4%)	9,565 (18%)	10,545 (19.7%)	11,883 (22.3%)	12,574 (23.4%)	12,625 (24.4%)	11,818 (23.5%)
250-400%	9,770 (15.4%)	12,177 (19.6%)	12,319 (20.1%)	12,668 (21.1%)	13,201 (21.8%)	11,236 (20.7%)	10,009 (18.9%)	10,050 (18.9%)	9,397 (17.6%)	14,871 (27.9%)	16,883 (31.4%)	14,639 (28.3%)	12,971 (25.8%)
>400%	31,977 (50.4%)	28,837 (46.4%)	28,581 (46.6%)	27,315 (45.6%)	26,865 (44.4%)	24,073 (44.4%)	23,678 (44.8%)	23,724 (44.7%)	23,577 (44.1%)	21,047 (39.5%)	19,086 (35.5%)	18,342 (35.4%)	17,876 (35.6%)
Unknown	14,499 (22.8%)	13,604 (21.9%)	12,726 (20.7%)	11,658 (19.4%)	11,570 (19.1%)	10,832 (20%)	9,973 (18.9%)	9,712 (18.3%)	9,945 (18.6%)	5,471 (10.3%)	5,277 (9.8%)	6,173 (11.9%)	7,601 (15.1%)

Race/ethnicity													
Asian	4,223 (6.7%)	4,556 (7.3%)	4,354 (7.1%)	4,495 (7.5%)	4,728 (7.8%)	4,480 (8.3%)	4,567 (8.6%)	4,534 (8.5%)	4,631 (8.7%)	4,725 (8.9%)	4,665 (8.7%)	3,853 (7.4%)	3,364 (6.7%)
Black	5,681 (9%)	5,848 (9.4%)	6,106 (9.9%)	5,750 (9.6%)	5,794 (9.6%)	4,482 (8.3%)	4,427 (8.4%)	4,425 (8.3%)	4,607 (8.6%)	4,638 (8.7%)	4,818 (9%)	4,424 (8.5%)	4,137 (8.2%)
Hispanic	7,941 (12.5%)	7,424 (11.9%)	7,610 (12.4%)	7,268 (12.1%)	7,406 (12.2%)	7,136 (13.2%)	7,149 (13.5%)	7,303 (13.8%)	7,341 (13.7%)	7,538 (14.2%)	7,567 (14.1%)	7,018 (13.6%)	6,390 (12.7%)
Unknown	6,935 (10.9%)	5,549 (8.9%)	4,548 (7.4%)	3,513 (5.9%)	2,759 (4.6%)	2,041 (3.8%)	1,535 (2.9%)	1,380 (2.6%)	1,392 (2.6%)	1,149 (2.2%)	1,409 (2.6%)	3,432 (6.6%)	5,419 (10.8%)
White	38,690 (61%)	38,788 (62.4%)	38,768 (63.2%)	38,938 (64.9%)	39,844 (65.8%)	36,081 (66.5%)	35,184 (66.6%)	35,409 (66.7%)	35,493 (66.4%)	35,222 (66.1%)	35,361 (65.7%)	33,052 (63.8%)	30,956 (61.6%)

Supplemental Exhibit 6. Forest plot of adjusted odds of PTSD diagnosis.

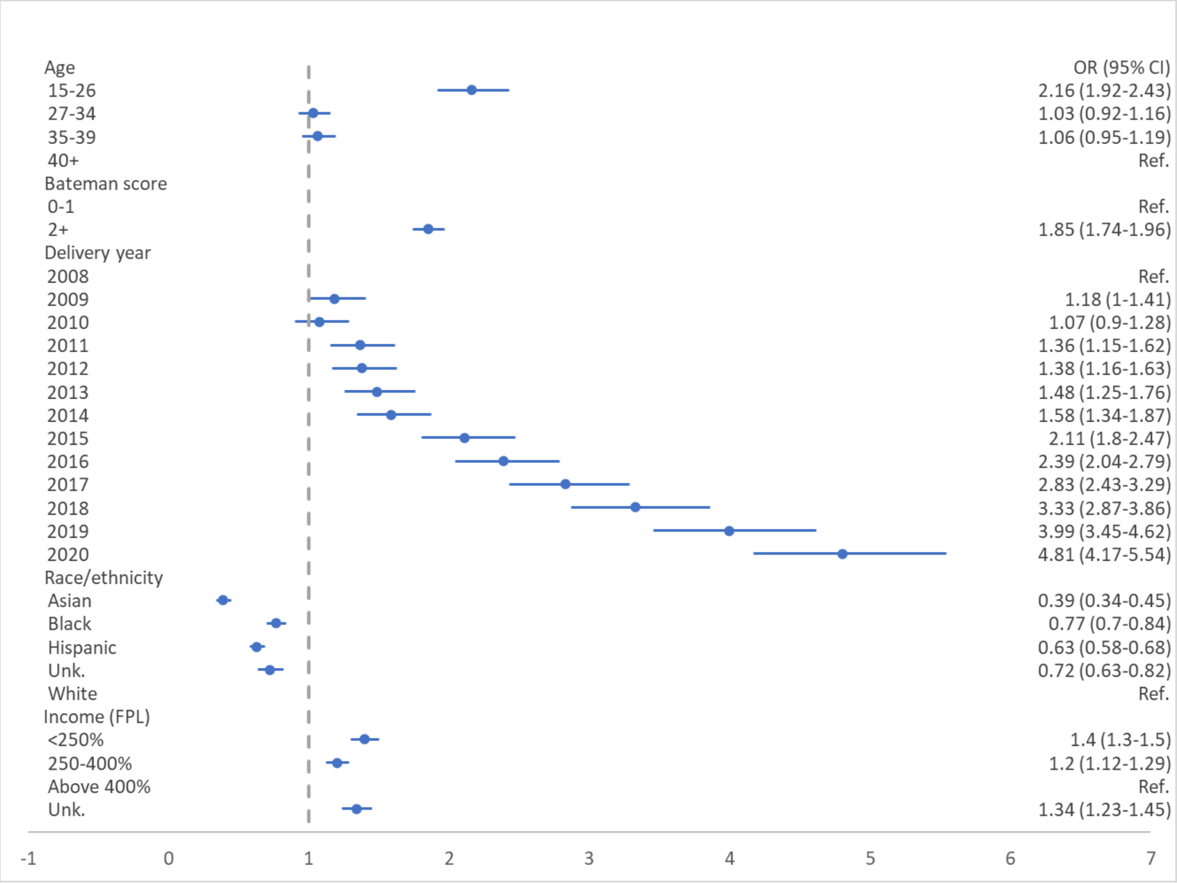

Supplemental Exhibit 7. Adjusted odds of PTSD diagnosis among those with and without PMAD.

	PMAD (n=140,084)			No PMAD (n=596,241)		
	Adjusted OR	95%CI	p-value	Adjusted OR	95%CI	p-value
Race/ethnicity						
Asian	0.94	(0.79-1.11)	0.4624	0.50	(0.38-0.66)	<0.0001*
Black	0.98	(0.89-1.09)	0.7156	0.84	(0.68-1.04)	0.1074
Hispanic	0.90	(0.81-0.98)	0.0219‡	0.63	(0.52-0.77)	<0.0001*
Unknown	0.92	(0.8-1.06)	0.269	0.63	(0.46-0.87)	0.0053‡
White	(ref)	(ref)	(ref)	(ref)	(ref)	(ref)
Year						
2008	(ref)	(ref)	(ref)	(ref)	(ref)	(ref)
2009	1.17	(0.95-1.43)	0.1481	1.08	(0.79-1.49)	0.6301
2010	1.18	(0.96-1.45)	0.1094	0.60	(0.41-0.88)	0.0081‡
2011	1.41	(1.15-1.72)	0.0007†	0.93	(0.67-1.3)	0.6796
2012	1.34	(1.1-1.63)	0.0041‡	1.01	(0.73-1.4)	0.9573
2013	1.46	(1.2-1.78)	0.0002†	0.98	(0.7-1.38)	0.9294
2014	1.47	(1.21-1.8)	0.0001†	1.20	(0.87-1.67)	0.2702
2015	2.00	(1.66-2.41)	<0.0001*	1.16	(0.83-1.61)	0.386
2016	2.10	(1.75-2.52)	<0.0001*	1.50	(1.1-2.05)	0.0103‡
2017	2.39	(2-2.86)	<0.0001*	1.56	(1.14-2.13)	0.005‡
2018	2.66	(2.23-3.16)	<0.0001*	1.71	(1.26-2.32)	0.0006†
2019	2.91	(2.45-3.45)	<0.0001*	2.32	(1.74-3.11)	<0.0001*
2020	3.20	(2.7-3.79)	<0.0001*	2.61	(1.96-3.49)	<0.0001*
Age						
15-26	1.87	(1.64-2.13)	<0.0001*	1.41	(1.04-1.89)	0.0249‡
27-34	1.01	(0.89-1.14)	0.8726	0.88	(0.66-1.15)	0.348
35-39	0.98	(0.86-1.11)	0.7393	1.12	(0.85-1.47)	0.4066
40+	(ref)	(ref)	(ref)	(ref)	(ref)	(ref)

OBCMI Score						
0-1	(ref)	(ref)	(ref)	(ref)	(ref)	(ref)
2+	1.48	(1.39-1.58)	<0.0001*	1.39	(1.19-1.62)	<0.0001*
Poverty						
Unknown	1.46	(1.33-1.6)	<0.0001*	1.02	(0.84-1.23)	0.8635
<250%	1.38	(1.28-1.5)	<0.0001*	1.06	(0.89-1.26)	0.4966
250-400%	1.23	(1.14-1.33)	<0.0001*	1.07	(0.92-1.25)	0.3776
>400%	(ref)	(ref)	(ref)	(ref)	(ref)	(ref)

Supplemental Exhibit 8. Forest plot of adjusted odds of PTSD diagnosis among those (a) with and (b) without PMAD.

(a) with PMAD

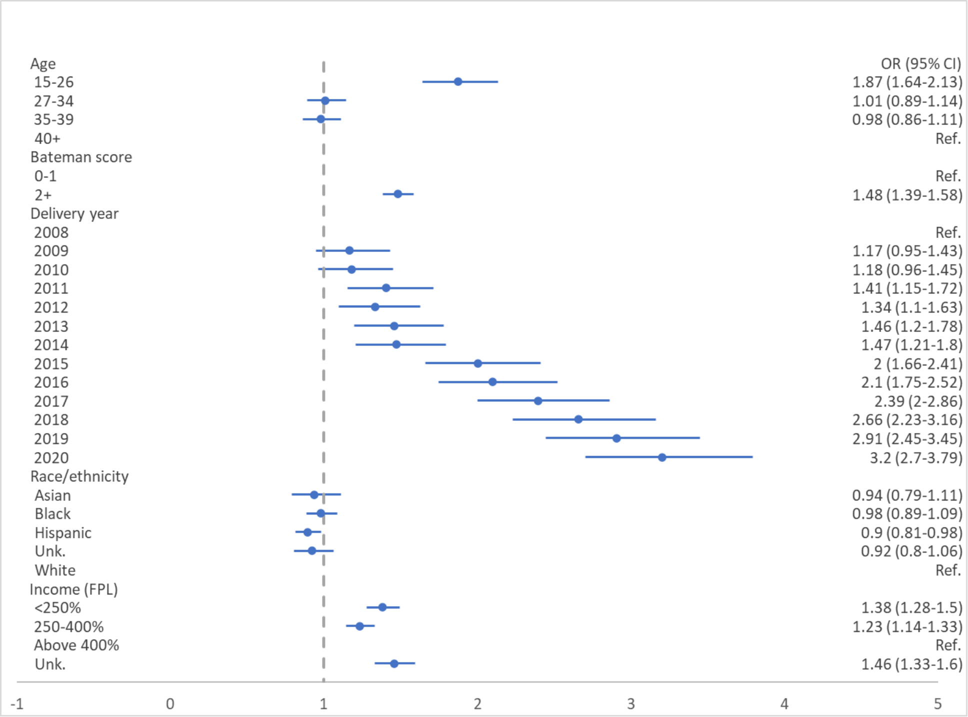

(b) without PMAD

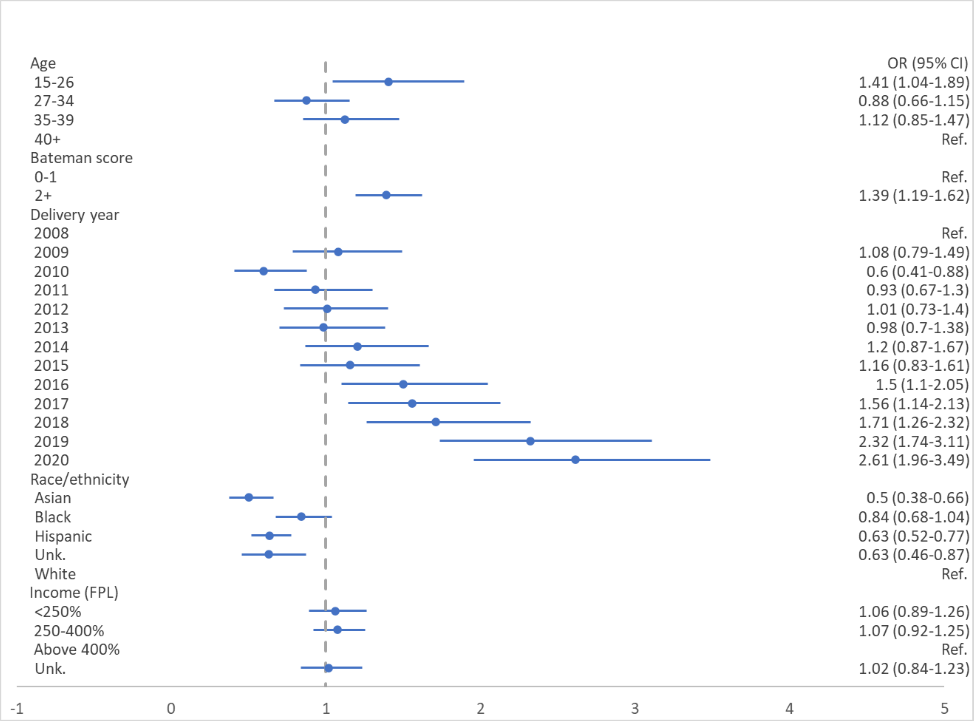

Technical Appendix

Multivariate Logistic Regression

Our unit of analysis was the delivery. We conducted a multivariable logistic regression with the dependent variable perinatal PTSD and the following independent variables: age, binary indicator for a Bateman score 2 or greater, race/ethnicity, federal poverty level, and year of delivery. We obtained predicted probabilities by year of delivery and race/ethnicity to assess disparities by race/ethnicity in adjusted trends in perinatal PTSD over time. Predicted probabilities were multiplied by 10,000 to produce rates per 10,000 deliveries.

We also ran the same model stratified by perinatal mood and anxiety disorder (PMAD) diagnoses.

N = 736,325 deliveries from 2008 to 2020

Exhibit 2. Unadjusted and adjusted odds of PTSD diagnosis among delivering individuals 2008-2020.

	**Odds ratio (95% confidence interval)**	**Estimate**	**Standard error**	***p*-value**
Delivery year				
2008	Ref.			
2009	1.18 (1-1.41)	0.1683	0.08821	0.0564
2010	1.07 (0.9-1.28)	0.07201	0.09046	0.426
2011	1.36 (1.15-1.62)	0.3111	0.086	0.0003
2012	1.38 (1.16-1.63)	0.3195	0.08535	0.0002
2013	1.48 (1.25-1.76)	0.3952	0.08601	<.0001
2014	1.58 (1.34-1.87)	0.4604	0.08548	<.0001
2015	2.11 (1.8-2.47)	0.7468	0.08072	<.0001
2016	2.39 (2.04-2.79)	0.8695	0.07898	<.0001
2017	2.83 (2.43-3.29)	1.0388	0.07724	<.0001
2018	3.33 (2.87-3.86)	1.2026	0.07549	<.0001
2019	3.99 (3.45-4.62)	1.3849	0.07411	<.0001
2020	4.81 (4.17-5.54)	1.5697	0.07288	<.0001
Age				
15-26	2.16 (1.92-2.43)	0.7696	0.06015	<.0001
27-34	1.03 (0.92-1.16)	0.03236	0.05727	0.572
35-39	1.06 (0.95-1.19)	0.06162	0.05767	0.2853
40+	Ref.			

Race/ethnicity				
Asian	0.39 (0.34-0.45)	-0.9444	0.07328	<.0001
Black	0.77 (0.7-0.84)	-0.2673	0.04651	<.0001
Hispanic	0.63 (0.58-0.68)	-0.4641	0.04313	<.0001
Unknown	0.72 (0.63-0.82)	-0.3286	0.06463	<.0001
White	Ref.			
OBCMI score				
0-1	Ref.			
2+	85 (1.74-1.96)	0.6144	0.03094	<.0001
Federal poverty level (FPL)				
<250%	1.4 (1.3-1.5)	0.3331	0.03595	<.0001
250-400%	1.2 (1.12-1.29)	0.1844	0.03463	<.0001
>400%	Ref.			
Unknown/missing	1.34 (1.23-1.45)	0.291	0.04156	<.0001

Supplemental Exhibit 7. Adjusted odds of PTSD diagnosis among those with PMAD.

	Odds ratio (95% confidence interval)	Estimate	Standard error	*p*-value
Delivery year				
2008	Ref.			
2009	1.08 (0.79-1.49)	0.1529	0.1057	0.1481
2010	0.6 (0.41-0.88)	0.168	0.1049	0.1094
2011	0.93 (0.67-1.3)	0.3412	0.1012	0.0007
2012	1.01 (0.73-1.4)	0.2893	0.1007	0.0041
2013	0.98 (0.7-1.38)	0.3782	0.101	0.0002
2014	1.2 (0.87-1.67)	0.3879	0.1011	0.0001
2015	1.16 (0.83-1.61)	0.6942	0.09475	<.0001
2016	1.5 (1.1-2.05)	0.7416	0.09326	<.0001
2017	1.56 (1.14-2.13)	0.873	0.09112	<.0001
2018	1.71 (1.26-2.32)	0.9772	0.08919	<.0001
2019	2.32 (1.74-3.11)	1.0667	0.08792	<.0001
2020	2.61 (1.96-3.49)	1.164	0.08646	<.0001
Age				
15-26	1.41 (1.04-1.89)	0.627	0.06665	<.0001
27-34	0.88 (0.66-1.15)	0.01022	0.06369	0.8726
35-39	1.12 (0.85-1.47)	-0.02147	0.06453	0.7393
40+	Ref.			
Race/ethnicity				
Asian	0.5 (0.38-0.66)	-0.06369	0.08667	0.4624
Black	0.84 (0.68-1.04)	-0.01901	0.05217	0.7156
Hispanic	0.63 (0.52-0.77)	-0.1104	0.04813	0.0219
Unknown	0.63 (0.46-0.87)	-0.07849	0.07102	0.269
White	Ref.			
OBCMI score				
0-1	Ref.			
2+	1.39 (1.19-1.62)	0.3935	0.03419	<.0001
Federal poverty level (FPL)				
<250%	1.06 (0.89-1.26)	0.3239	0.03992	<.0001
250-400%	1.07 (0.92-1.25)	0.2108	0.03899	<.0001
>400%	Ref.			
Unknown/missing	1.02 (0.84-1.23)	0.3764	0.04642	<.0001

Supplemental Exhibit 7. Adjusted odds of PTSD diagnosis among those without PMAD.

	**Odds ratio (95% confidence interval)**	**Estimate**	**Standard error**	***p*-value**
Delivery year				
2008	Ref.			
2009	1.17 (0.95-1.43)	0.1529	0.1057	0.1481
2010	1.18 (0.96-1.45)	0.168	0.1049	0.1094
2011	1.41 (1.15-1.72)	0.3412	0.1012	0.0007
2012	1.34 (1.1-1.63)	0.2893	0.1007	0.0041
2013	1.46 (1.2-1.78)	0.3782	0.101	0.0002
2014	1.47 (1.21-1.8)	0.3879	0.1011	0.0001
2015	2 (1.66-2.41)	0.6942	0.09475	<.0001
2016	2.1 (1.75-2.52)	0.7416	0.09326	<.0001
2017	2.39 (2-2.86)	0.873	0.09112	<.0001
2018	2.66 (2.23-3.16)	0.9772	0.08919	<.0001
2019	2.91 (2.45-3.45)	1.0667	0.08792	<.0001
2020	3.2 (2.7-3.79)	1.164	0.08646	<.0001
Age				
15-26	1.87 (1.64-2.13)	0.627	0.06665	<.0001
27-34	1.01 (0.89-1.14)	0.01022	0.06369	0.8726
35-39	0.98 (0.86-1.11)	-0.02147	0.06453	0.7393
40+	Ref.			
Race/ethnicity				
Asian	0.94 (0.79-1.11)	-0.06369	0.08667	0.4624
Black	0.98 (0.89-1.09)	-0.01901	0.05217	0.7156
Hispanic	0.9 (0.81-0.98)	-0.1104	0.04813	0.0219
Unknown	0.92 (0.8-1.06)	-0.07849	0.07102	0.269
White	Ref.			
Bateman score				
0-1	Ref.			
2+	1.48 (1.39-1.58)	0.3935	0.03419	<.0001
Federal poverty level (FPL)				
<250%	1.38 (1.28-1.5)	0.3239	0.03992	<.0001
250-400%	1.23 (1.14-1.33)	0.2108	0.03899	<.0001
>400%	Ref.			
Unknown/missing	1.46 (1.33-1.6)	0.3764	0.04642	<.0001
